# Collagen Alignment via Electro-Compaction for Biofabrication Applications: A Review

**DOI:** 10.3390/polym14204270

**Published:** 2022-10-12

**Authors:** Benjamin P. Carr, Zhi Chen, Johnson H. Y. Chung, Gordon G. Wallace

**Affiliations:** Australian Research Council Centre of Excellence for Electromaterials Science, Intelligent Polymer Research Institute, University of Wollongong, Wollongong, NSW 2522, Australia

**Keywords:** collagen, alignment, electro-compaction, electro-chemical alignment

## Abstract

As the most prevalent structural protein in the extracellular matrix, collagen has been extensively investigated for biofabrication-based applications. However, its utilisation has been impeded due to a lack of sufficient mechanical toughness and the inability of the scaffold to mimic complex natural tissues. The anisotropic alignment of collagen fibres has been proven to be an effective method to enhance its overall mechanical properties and produce biomimetic scaffolds. This review introduces the complicated scenario of collagen structure, fibril arrangement, type, function, and in addition, distribution within the body for the enhancement of collagen-based scaffolds. We describe and compare existing approaches for the alignment of collagen with a sharper focus on electro-compaction. Additionally, various effective processes to further enhance electro-compacted collagen, such as crosslinking, the addition of filler materials, and post-alignment fabrication techniques, are discussed. Finally, current challenges and future directions for the electro-compaction of collagen are presented, providing guidance for the further development of collagenous scaffolds for bioengineering and nanotechnology.

## 1. Collagen

**Figure 1:**
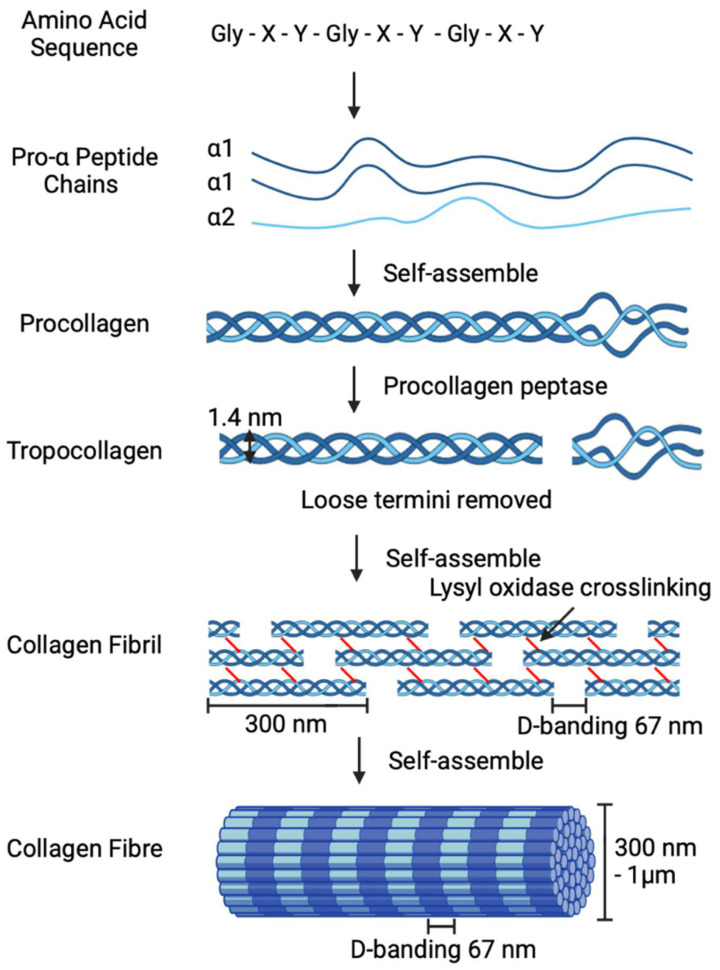
Schematic of collagen type I synthesis including amino acid sequence, pro-α peptide chains, procollagen, tropocollagen, collagen fibril, and fibre.

Collagen is the most abundant protein in animals, contributing up to 30% of the major structural components of the extracellular matrix [1,2]; it has been widely explored for biofabrication and tissue engineering applications. Some biofabrication-based applications of collagen have included bone, cartilage, tendons, muscles, trachea, oesophagus, blood vessels, and corneas [3,4,5]. Currently, 28 known subtypes of collagen have been identified by its primary amino acid structure and from genetic sequencing [6]. Type I collagen is the most common subtype, comprising over 90% of collagen in the body [7], which is found in skin, bones, tendons, ligaments, blood vessels, and organs [5]. Collagen type I has comparatively the simplest structure of the collagen protein family. The biopolymer is formed by repeating tripeptide sequences: “Glycine-X-Y”, where X and Y are mainly proline and hydroxyproline, forming polypeptide chains (Figure 1) [8]. Additional collagen types are named using roman numerals in the order of their discovery (II to XXVIII) and are categorised by their structure (Table 1). There was thought to be an epidermal collagen type XXIX; however, subsequent genetic sequencing determined that type XXIX (COL29A1 gene) was genetically identical to type VI (COL6A5 gene) and the α1(XXIX) chain corresponded to the α5(VI) chain [6,9]. 

For all types of collagen, amino acid chains self-assemble to form polypeptide α-helix chains, and three α-helix chains (e.g., type I, two α1(I), and one α2(I)) self-assemble to form a triple helix structure, termed procollagen (Figure 1) [10,11]. Procollagen is linked via hydrogen bonds and has an approximate diameter of 1.4 nm and a length of 300 nm [12]. *Procollagen peptidase* cleaves the N- and C-terminals from the precursor procollagen, forming tropocollagen. Tropocollagen will self-assemble via fibrillogenesis to form fibrils and, subsequently, collagen fibres [4,13]. Collagen fibrils self-assemble to create a highly organised quarter-stagger package pattern. The distance between longitudinally aligned tropocollagen helices is 67 nm, known as D-banding [4,12]. The final fibre diameter can vary from 300 nm–1 μm with alternated 67 nm D-banding along the fibre [5,14]. 

One major limitation of conventional collagen gels is their suboptimal mechanical properties compared to natural tissues. Specifically, conventional unaligned collagen gels have a Young’s modulus range of 10–400 kPa [15,16,17], an ultimate tensile strength of 4 kPa, and a strain of 70% [18]. In comparison, applications such as aorta biofabrication require an approximate Young’s modulus of 1.0 MPa, an ultimate tensile strength of 1.2 MPa, and a strain of 1.1 MPa [19], which shows that conventional collagen scaffolds are insufficient mechanically. Due to the difference between the achieved and desired mechanical properties when using conventional collagen gels, several techniques have been reported to strengthen conventional collagen gels, primarily consisting of crosslinking. Collagen crosslinking methods are categorised into chemical, physical, and enzymatic, or combinations, such as photocrosslinking (chemical modification and ultraviolet light) [20]. 

**Figure 2:**
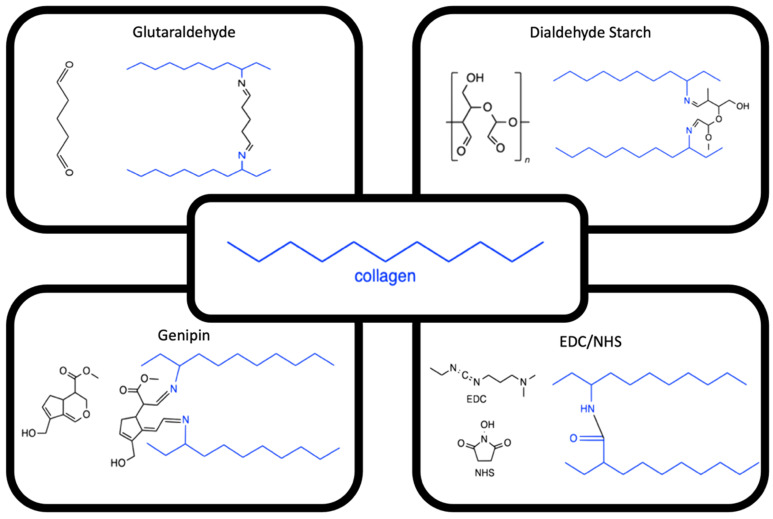
Collagen chemical crosslinker mechanisms glutaraldehyde, dialdehyde starch, genipin, and EDC/NHS [37,41,46,47].

The most frequently utilised crosslinking method is chemical crosslinking; this involves introducing interfibrillar connections between the collagen fibres [27]. Chemical crosslinking agents routinely used with collagen scaffolds include glutaraldehyde [28], dialdehyde starch [29,30], genipin [31,32,33], and ethyl(dimethylaminopropyl)carbodiimide/N-hydroxysuccinimide (EDC/NHS) [34,35] (Figure 2). Glutaraldehyde is a straight-chain saturated dialdehyde with five carbons [28], whilst dialdehyde starch is a polysaccharide derived from modified starch [36]. Both Glutaraldehyde and dialdehyde starch function by two highly reactive aldehydic groups forming covalent bonds with free amine groups on adjacent collagen peptide chains [28,37]. Glutaraldehyde is noted to be one of the most effective crosslinkers; however, unreacted molecules result in cytotoxic responses [38] when compared to dialdehyde starch, which has good biocompatibility [39]. Genipin is naturally found in *Genipa americana* fruit extract, with lower toxicity compared to Glutaraldehyde [40]. During the crosslinking process, the free amine group of collagen acts as a nucleophile to open the genipin ring and form a covalent bond with the olefinic carbon, resulting in an unstable intermediate. During the second stage of the reaction, the genipin intermediate aldehyde group is attacked by the amine group of a different collagen fibril, resulting in a second collagen fibril being covalently bound and completing the crosslink [41]. EDC/NHS is a ‘zero-length’ crosslinking method which activates collagen molecules to directly form bonds between adjacent fibrils [20]. EDC/NHS successfully mimics enzymes, such as *Lysyl oxidase*, that naturally crosslink and stabilise collagen [42] whilst remaining bio-compatible and non-cytotoxic [43]. Crosslinking occurs in several steps: first, EDC binds to the carboxylic acid when collagen forms an o-acylisourea intermediate; next, NHS binds, and urea is released; lastly, the EDC/NHS-prepared collagen molecule bonds to the amine group, resulting in a stable amide bond between adjacent collagen molecules [44,45]. 

Physical crosslinking methods utilise high (>90 °C) or low (<−50 °C) temperatures to dehydrate samples whilst under a vacuum to compress the scaffolds, resulting in dehydrothermal crosslinking [48,49] or freeze-drying, respectively [50,51]. When temperature and vacuum are applied, the carboxylic and amine groups form bonds and release water; in the case of freeze-drying, the water is trapped, forming a porous scaffold [45,52]. Enzymatic crosslinking uses enzymes such as *lysyl oxidase* [53] or *transglutaminase* [54], which modify the amino groups, resulting in fibrillar bonds.

Finally, photo-crosslinking is a combination of chemical and physical crosslinking [55]. Methacrylated collagen is prepared from methacrylic anhydride monomers undergoing nucleophilic substitution with the lysine residues in the collagen [56]. When short-wavelength ultraviolet is applied, direct excitation of the acrylic double bond occurs, resulting in crosslinking [57]. Riboflavin (vitamin B_2_) crosslinking is induced by singlet oxygen generation from ultraviolet-A excited riboflavin, resulting in covalent bond formation between the amino acids of the collagen fibrils [58]. Compared to conventional photo-initiators, riboflavin has low cytotoxicity [59]. 

## 2. Overview of Collagen Alignment Techniques

**Figure 3:**
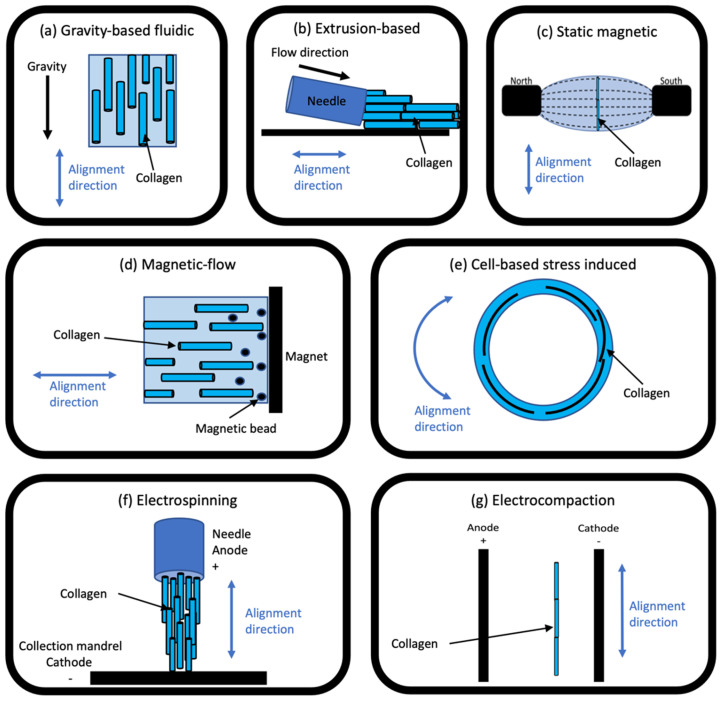
Schematic of collagen alignment methods: (**a**) gravity-based fluidic, (**b**) extrusion-based, (**c**) static magnetic, (**d**) magnetic-flow, (**e**) cell-based stress induced, (**f**) electrospinning, and (**g**) electro-compaction.

Another method of increasing collagen-based scaffold strength is through achieving anisotropic alignment. Anisotropically aligned collagen-based structures are observed throughout the body in tissues such as tendons, muscles, nerves, intervertebral discs [60], blood vessels [61,62], and corneas [35]. Ex vivo alignment aims to mimic the tissue’s natural fibre direction, contributing to the tissue’s ability to withstand physiological loads and mechanical stressors [63]. Another advantage of aligned-collagen-based scaffolds is that they provide biophysical cues to direct cell adherence, migration, and proliferation [64]. Collagen alignment can be achieved by several methods, namely, gravity and extrusion-based fluidic alignment [65], static magnetic alignment [66], magnetic-flow alignment [11], cell-based stress-induced self-alignment [67], electrospinning [68], and electrophoretic-based electro-compaction (EC; Figure 3) [69]. The principles and recent advances are introduced in this review, with discussion on the advantages and disadvantages of each method, including a sharper focus on EC. This information can contribute to producing mechanically robust biomimetic scaffolds for various biofabrication applications. 

The **gravity-based fluidic alignment** of collagen is comparatively the simplest method, as first reported by Elsdale and Bard in 1972 [70]. A solution of collagen containing Eagle’s medium and NaOH (pH = 7.6) was poured into a mould at an angle. The solution was simultaneously incubated whilst allowing the solution to drain and undergo fibrillogenesis. The resulting scaffold demonstrated collagen fibrils aligned parallel to the direction of flow [70,71]. 

Utilising the fundamental principles of **fluidic alignment**, moving the collagen whilst it underwent fibrillogenesis allowed an extrusion-based technique to be developed by Kirkwood and Fuller in 2009 [65]. Briefly, a custom three-axis arm printing device was used to extrude a collagen solution through a needle that was parallel to the printing surface. The collagen was aligned parallel to the deposition direction on the edges, whilst the middle showed isotropic alignment. The inconsistent alignment throughout the film was identified as a significant limitation. Further work implemented a flattened needle, allowing for consistent alignment throughout the structure. For the application of bone biofabrication, primary osteoblasts were seeded on aligned substrates, resulting in cells exhibiting preferential alignment along the fibre orientation [72]. The technique has been also investigated for applications such as blood vessels fabrication [73] and cochlear implant coatings [74]. 

Early reported uses of **static magnetic alignment** from the late 1970s to the 2000s consisted of exposing collagen to magnets in the range of 1.9–9.4 T [75,76,77,78,79,80]. When collagen is deposited within the magnetic field, fibrils align perpendicular to the field and magnet [81]. Initially, there was a large amount of variation in the parameters for this method of alignment. In 2007, Torbet et al. developed a more standardised approach [82], which was widely used until 2010 [83]. The method consisted of collagen aliquots being loaded into cooled glass-bottom plastic culture dishes and placed parallel to the magnet. The magnetic field (7 T) was applied for 30 min whilst the temperature was increased to 20 °C, resulting in collagen gelation [82]. Since its first use, the technique has been modified to use stronger magnets (12 T) [81] and, most recently, in 2021, further modified to include a superconductor magnet (13 T) [66]. The use of stronger magnetic fields induces quicker and more dense alignment [84]. Mechanical characterisation of collagen-based magnetic alignment demonstrated a linear compressive modulus between 7–21 kPa and storage modulus between 26–75 Pa. Both moduli were found to increase with increasing polymerisation temperatures. Over the decades, magnetically-aligned collagen-based scaffolds have been utilised for clinical applications such as cornea [82], bone [76], tendon [66,79], and nerve tissue biofabrication [77]. Initially, for corneal applications, keratocytes were used to assess the cellular response to the magnetically-aligned scaffolds. This demonstrated that the cells seeded on the aligned scaffolds uniformly orientate in the direction of the collagen fibrils, whereas, in contrast, the unaligned scaffolds remained randomly distributed [82]. Further work investigated nerve regeneration, where tubes filled with collagen and Schwann cells (harvested from male Wistar rats) were subjected to a magnetic field (8T for 2 h) and then implanted between the severed ends of the rat’s sciatic nerves. The study demonstrated that static magnetically-aligned collagen can promote nerve regeneration and recovery in neurological functioning [85]. 

The method of **magnetic-flow alignment** combines the principles from both magnetic and fluidic alignment. Guo and Kaufman, in 2007, aimed to simplify the previously described magnetic alignment process, as the previous methods required specialised equipment that remained widely inaccessible [11]. The new method utilises magnetic beads (diameter 2.5 µm) that are mixed into a solution of collagen at 4 °C. The solution is subsequently placed on a glass microscope slide with a coverslip followed by a small magnet (such as a metal stir bar) and is incubated. As a result of this work, both thick (several mm) and thin (10–20 µm) collagen scaffolds with highly orientated fibrils were produced. Interestingly, it was confirmed that the mechanism of alignment was a combination of both flow (due to bead movement) and the magnetic field. When either variable is removed, the scaffolds did not align [11,86]. When crosslinked with genipin (0.25%), the scaffolds possessed a tangent modulus of approximately 1 MPa and a strain of 0.035%, higher than that of conventional collagen gels [87]. Furthermore, glioma cells (C6) were successfully incorporated at the same time as the magnetic beads and aligned in the presence of the cells. Initially, the cells took several hours to spread through the aligned collagen, which is consistent with isotropic scaffolds; however, once the cells spread, they align on the aligned scaffold [11]. 

The in vitro alignment of collagen via organised **cell-based stress-induced self-alignment** relies on the alignment occurring during collagen synthesis. The main benefit of cell-based alignment is that the collagen is not subjected to extraction and purification processing, which has been shown to affect the properties of collagen-based scaffolds [5,13,67]. The method had previously utilised the addition of stress-shielding exogenous proteins and polymers paired with the application of external forces to promote the alignment [67,88,89,90]. Schell et al. investigated a method of alignment using the innate stress of different geometries without further additives. Various shaped moulds were seeded with human dermal fibroblasts (hDFs) and cultured for four weeks, allowing for collagen synthesis. The results showed a successful alignment using the toroid mould, forming circumferentially aligned scaffolds [91]. In 2018, Wilks et al. further used this method and decellularised the scaffolds on day 14 of culturing, producing circumferentially-aligned collagen scaffolds [67]. The aligned scaffolds were later re-cellularised with hDFs, and, after 12 h, the cells had attached with typical fibroblast morphology and orientated circumferentially in the direction of the alignment. The mechanical properties of this method have not been investigated; however, it remains a promising, completely cell-based alignment method that relies on the development of cell-mediated tension. 

**Electrospinning** is the process of fibre alignment via a combination of electrophoretic and extrusion-based techniques. The technique, first reported over a century ago, has since been widely used with various synthetic polymers [92]. Collagen alignment via electrospinning was reported in 2002 by Matthews et al. [93]. The process consists of dissolving collagen in a solvent, such as a fluoroalcohol (1,1,1,3,3,3-hexafluoro-2-propanol; HFP or 2,2,2-trifluoroethanol; TFE) [68]. A syringe pump then extrudes the solution through a small gauge needle connected to a high-voltage power supply. The collagen thread is then collected onto a conductive metal plate or mandrel [94]. The resulting threads can range in diameter from 10 nm to a few microns and can be controlled by altering the processing parameters. Variables related to processing parameters include voltage, extrusion speed, and needle-ground distance, whilst solution properties, such as collagen source, solvent, and concentration and environmental conditions, can affect electrospinning [68,92,94,95]. One of the primary benefits of electrospun collagen fibres is their enhanced mechanical properties when compared to conventional collagen scaffolds. Specifically, the shear modulus of dry uncrosslinked threads was 29 MPa, whilst glutaraldehyde vapour-crosslinked threads achieved 48 MPa, and the wet crosslinking was 5.2 MPa [96]. Electrospun collagen threads have been used for various applications including skin, wound healing [97,98,99,100], nerve regeneration [101,102,103,104], blood vessel [105,106], muscle [107,108], and bone [109,110] fabrication. 

**Electro-compaction** (EC) is an electro-chemical process of collagen alignment where isoelectric focusing and the generation of a pH gradient are utilised [111]. The process had remained largely unexplored since the 1970s when Marino et al. applied an electrical current to a solution of collagen. However, the technology at the time could not accurately determine fibre orientation and, thus, the alignment of the collagen fibres. Due to the technological limitations, it was concluded that there were random regions of preferential alignment, but no overall discernible ‘grain’ [112] or D-banding was present [113]. This was due to an inability to accurately observe the collagen alignment such that it was considered ineffective and remained further unexplored until 2008 [69]. In principle, a solution of collagen is loaded between two electrodes and a current is applied; this results in a scaffold in the form of a mechanically robust highly orientated thread or membrane, which is determined by electrode shape. EC collagen scaffolds have been used to fabricate various scaffolds for a range of tissue types and clinical applications, including cornea [18,35], muscle [114], tendon [64], nerve [115], skin [116], and blood vessels [61,117]. 

Collagen is a natural biopolymer that has been widely investigated as a biomaterial due to its natural prevalence and good cytocompatibility. However, collagen-based scaffolds alone lack the mechanical properties required by many tissues; therefore, methods to increase the mechanical characteristics have been explored but have previously been limited, primarily due to crosslinking. The anisotropic alignment of collagen is able to enhance scaffold strength whilst better mimicking natural tissue orientation and improving cellular activity. As discussed, there are several methods to achieve the alignment of collagen, each with its associated advantages and limitations (Table 2). 

The degree of alignment and achievable scaffold shapes varies from method to method. Cell-based alignment demonstrates the least amount of circumferential alignment due to its reliance on fibrinogenesis via the fibroblasts. Similarly, extrusion-based alignment is partially aligned with noted inconsistency throughout the thread. In contrast, EC and static magnetic alignment can successfully form highly aligned and dense scaffolds in multiple shapes. The amount of collagen required can be categorised into low (<5 mg.mL^−^^1^) and high (>15 mg.mL^−^^1^); however, commercially-sourced collagen comes in a stock concentration of ~6–10 mg.mL^−^^1^. Due to the commercial availability, lower concentration requirements, as used in static, flow, magnetic, and EC methods, are optimal. Another major consideration is the effect of the alignment method on the structure of collagen. Specifically, electrospinning uses harmful solvents which are noted to dissolve collagen peptides, prevent reassembly, and cause D-band formation, resulting in denaturation and gelatinisation [96,118,119,120,121], whilst magnetic flow uses magnetic beads which remain in the scaffold after alignment, making these less ideal. It remains challenging to directly compare the mechanical properties of each alignment technique due to inconsistencies in the testing methods. However, a greater number of aligned scaffolds, such as EC, magnetic, or electrospun scaffolds, will possess higher mechanical properties compared to less aligned methods like gravity- or cell-based alignment. Collagen is already a good biomaterial with good biocompatibility, but its alignment was able to further improve overall cellular responses. Specifically, scaffolds seeded with various cell types have consistently demonstrated an increase in cell attachment and proliferation compared to conventional unaligned scaffolds. Finally, ease and time of processing are both considerations when thinking about quick and simple methods such as EC and magnetic flow, which provide a widely available method of alignment. In contrast, cell-based alignment requires a comparatively long time but does benefit from the collagen not undergoing extraction processing. Other methods, like static magnetic alignment, require specialised equipment, such as a superconductor magnet. Overall, EC has been identified as a simple and highly effective alignment method; however, no in-depth review has been undertaken and is the further focus of this review. 

## 3. Electro-Compaction

**Figure 4:**
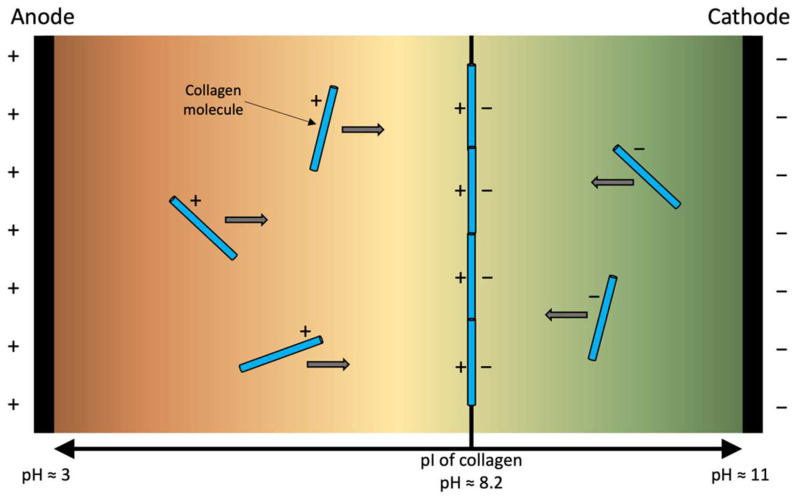
Schematic of collagen electro-compaction, illustrating the anode and cathode with associated charges and generated pH gradient, and demonstrating the charges gained by the collagen molecules and the aggregation at the isoelectric point (pI), where the net charge is 0.

The method for EC consists of an electrical current being applied between two electrodes across a solution of collagen, generating a pH gradient (anode pH ≈ 3 and cathode pH ≈ 11) and charging the collagen molecules (Figure 4) [18,61,120]. The collagen nearer the anode (positive electrode) gains a positive charge, whilst those nearer the cathode are charged negatively [120]. The combination of the pH gradient and charged collagen produces a highly organised anisotropically-aligned aggregation at the isoelectric point. The isoelectric point (pI) of collagen varies depending on the source, but for bovine hide, it is approximately at a pH = 8.2, where the net charge is 0 (Equation (1)) [13,120].

Anode: 2H_2_O − 4e^−^ → 4H^+^ + O_2_; Cathode: 4H_2_O + 4e^−^ → 4OH^−^ +2H_2_ (1)

Due to the specific pI of collagen, the aligned scaffold is formed between the electrodes, favouring the cathode with the collagen fibres parallel to the electrodes [69]. In addition to the alignment, the packing density of collagen increases from 5 mg.cm^−3^ non-EC to 50 mg.cm^−3^ post-treatment [120]. This also makes the concentration of EC collagen ~1030 mg.mL^−1^ [122,123,124,125] 17 times denser than non-EC conventional collagen gels. The optimal EC parameters, such as concentration, voltage, current density, and time, are generally specific to the application and desired outcome. However, higher voltage, closer electrodes, and collagen concentration have different optimal alignment times [126,127]. Specifically, 3 volts for 45 min [18] and 40 volts for 10 s [34] have both been successfully utilised. 

### 3.1. Sources of Collagen for Electro-Compaction

Several different sources of collagen have been used for EC, with the most common being type I, extracted from bovine hide. Other collagen sources used for EC have included porcine, fish, and rats. However, additionally, collagen has been successfully extracted from other sources such as human [128], ovine [129], equine [130], avian [131], and various marine (mammals and fish) [132,133]. The extraction method alters the structure and consists of, namely, chemically produced procollagen/telocollagen and enzymatically digested tropocollagen/atelocollagen [134,135]. Furthermore, each source of collagen has numerous subtypes of collage that exist in different tissues and aid the specific tilized functions within the body [5,13]. Different sources, types, and extraction methods possess various characteristics [13,136,137], such as viscosity [138,139], isoelectric point (pI) [140], and molecular weight [141,142], which ultimately allows for the ability to control the scaffold properties and EC processing.

### 3.2. Electro-Compacted Scaffold Types

**Figure 5:**
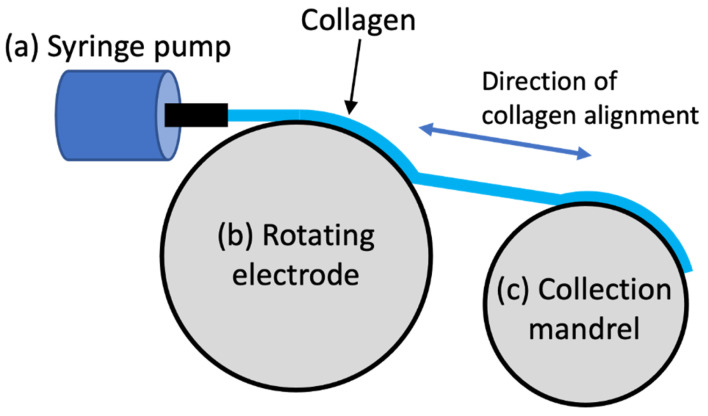
Schematic of rotating electrode electro-chemical alignment device (REEAD) for continuous electro-compaction of threads, (**a**) syringe pump, (**b**) rotating wheel with electrodes on the edge and collagen between, and (**c**) rotating collection mandrel.

Over recent years, progressively more complex EC collagen scaffolds have been utilized for biofabrication applications, commencing in 2008 with the generation of simple threads using linear electrodes [69] and membranes with planar electrodes [111]. Membranes have since been put into various shapes via EC, determined by the shape of the spacer or mould between the electrodes. Such examples have included rectangular, round [35], and irregular hexadecagon [114] shaped membranes. More recently, concentric tube electrodes have been used to fabricate tubular scaffolds [61], and curviplanar electrodes have been utilised to fabricate domes [120]. Initially, the length of the aligned collagen threads was restricted to the length of the linear electrodes. Younesi et al. addressed this limitation by developing a device for the continuous EC of collagen threads called REEAD (rotating electrode electro-chemical alignment device, Figure 5). The REEAD device utilises a syringe pump to extrude collagen onto the first of two rotating wheels [143]. The first wheel contains two parallel electrodes, as is used in a regular EC setup. Collagen is extruded between the electrodes whilst the wheel is in motion. Depending on the parameters, mainly speed and voltage, different thicknesses of collagen threads can be achieved (0.10–0.15 mm) [34,144]. At the same time, the collection mandrel rotates proportionally to the electrode to collect the aligned thread. 

The mechanical properties of EC collagen scaffolds vary depending on the scaffold shape and preparation process; in particular crosslinking and the use of filler materials. Additionally, there is no standardised method for the mechanical testing of EC collagen scaffolds, thus resulting in some samples being desiccated prior to testing whilst others remained hydrated, leading to the inconsistency of the tests performed and reported. It is well established that EC collagen has superior mechanical properties compared to conventional collagen gels. Specifically, the ultimate tensile strength of EC collagen is 6.2 MPa, compared to conventional collagen <10 kPa [120]. Young’s modulus has been shown to increase this from ~1 MPa (when unaligned) to ~50 MPa after EC [120]. 

Table 3 summarises all reported mechanical characterisations of EC collagen scaffolds. However, for many applications, these mechanical properties remain suboptimal compared to natural tissues. The mechanical properties of natural tissues are highly varied and determined by the region-specific physiological load. For example, tendons have a Young’s modulus in the range of 1.0–1.5 GPa and an ultimate tensile strength of 100–140 MPa [145], whilst myocardium has a Young’s modulus in the range of 0.2–0.5 MPa and an ultimate tensile strength of 3–15 kPa [146,147]. One approach to creating more mechanically robust scaffolds is the addition of fillers. 

### 3.3. Enhancing Electro-Compacted Collagen Strength

After the alignment of the collagen, further steps can be used to increase scaffold strength, such as phosphate-buffered saline (PBS) treatment or crosslinking. Uquillas et al. investigated the mechanical effects of immediate post-alignment incubation and PBS treatment to promote fibrillogenesis. The results demonstrated that 1 × PBS incubated for 12 h produced mechanically competent threads with D-banding similar to the native tendons. Specifically, the ultimate tensile stress was 0.4 MPa, the strain was 100%, and Young’s modulus was 0.4 MPa [122]. However, despite 12 h being the identified period for optimal mechanics, methodologies from subsequent studies have commonly used 4–6 h [18,31,61,64,114,115,116,117,143]. Furthermore, EC collagen crosslinking has been limited to exclusively chemical crosslinkers, as summarised in Table 4. Briefly, EDC/NHS has been used at various concentrations with varying solvents with a treatment time of 4 h at room temperature, whilst genipin has been optimised at a concentration of 0.625% dissolved in 90% ethanol and incubated for 72 h [148]. 

Additionally, non-fibre-forming structural molecules, mainly glycosaminoglycans (GAG) and proteoglycans, have been used to enhance the scaffold strength, functioning similarly to a crosslinker [153]. Paderi and Panitch synthesised a dermatan sulphate-peptide sequence (DS-SILY) which mimics the natural structure and function of decorin, a small leucine-rich proteoglycan (SLRP) [154]. The leucine-rich protein core binds to the D-bands on the collagen fibrils and the dermatan sulphate glycosaminoglycan chain, followed by the binding of the chains to adjacent molecules to form inter-fibrillar crosslinks. When incorporated into EC collagen, the ultimate tensile strength increased to 1.5 MPa (Col:DS-SILY 1:30) [149]. 

### 3.4. Co-Electro-Compaction of Collagen with Fillers

As previously established, collagen has excellent biocompatibility, and when it is aligned, it has better mechanical properties. However, the mechanical strength remains suboptimal for many biofabrication applications. The use of additional materials, termed fillers, can be utilised to reinforce collagen-based scaffolds. The methods of filler incorporation are grouped into two main techniques: (a) homogenous co-electro-compaction (Co-EC) and (b) post-EC fabrication methods. Co-EC involves the homogenous incorporation of fillers into the collagen and then applying a current. Examples of materials suited for Co-EC are biopolymers, such as elastin [61], or polysaccharides, such as nanocellulose [31]. The primary determining factor of this method is the isoelectric point of the filler in relation to collagen (pH ≈ 8.2). Both materials are required to have similar isoelectric points allowing for isoelectric focusing on the same point. On the contrary, if the isoelectric point between the materials is too great, the materials will separate during EC. Thus, forming two independent scaffolds, each aligned at their respective isoelectric points [155]. 

Similar to collagen, **elastin** is a protein that forms part of the extracellular matrix. However, it is responsible for the elastic properties in tissues, specifically, stretching and contraction [156]. Nguyen et al. initially investigated the effects of soluble versus insoluble elastin when incorporated into EC collagen threads for application in small-diameter blood vessels [117]. Briefly, solutions of elastin (soluble or insoluble, 200 mg.mL^−1^) were mixed with collagen (3.1 mg.mL^−1^) at a ratio of 40:60 (*w*/*w*%). The solutions were subjected to Co-EC using stainless steel wire electrodes at 3 volts for 30 min, and then incubated (37 °C) in PBS for 6 h. Mechanical testing showed a decrease in Young’s modulus, ultimate tensile stress, and strain with the incorporation of elastin (Table 5). However, in vitro characterisation using rat aorta smooth muscle cells (rSMCs) and real-time polymerase chain reaction (PCR) determined a positive effect on the contractile phenotype of the cells when elastin was incorporated. Additionally, it was determined that the cells could sense the composition and topography of Co-EC fibres. A direct comparison between soluble and insoluble elastin determined that insoluble was better suited for biofabrication-based applications. This method was used again with insoluble elastin, with the ratio of collagen to elastin adjusted to 50:50 (*w*/*w*) [61]. 

**Nanocellulose** is a polymer sourced from the cellulose found in plants, bacteria, algae, and animals [157] and comes in two primary forms. First, nanostructured materials, including microcrystals and microfibrils, whilst the second are nanofibers such as nanofibrils, nanocrystals, and bacterial cellulose [158]. Nanocellulose has been used in various applications, including wound healing, blood vessel, corneal, heart valve, urethra, bone, and cartilage biofabrication [159]. The wide use of nanocellulose is due to its increased mechanical properties, biocompatibility, and low cytotoxicity [160]. As previously described, one primary consideration when choosing a material for Co-EC is the isoelectric point of the two materials. Cudjoe et al. modified the isoelectric point of TEMPO (2,2,6,6-Tetramethylpiperidine 1-oxyl)-oxidised cellulose nanocrystals (t-CNC), resulting in t-CNC−COOH, which favours the anode, whilst t-CNC−COOH^27^−NH_2_^73^ favoured the cathode at a pH of 7 [31]. The modification of nanocellulose allowed for successful Co-EC with collagen. The Co-EC utilised two parallel wire electrodes, and 20 volts were applied for 30 s. It was found that t-CNC−COOH^27^−NH_2_^73^ at 5% (*w*/*w* %) with collagen was optimal to increase strength when fabricating threads (Table 5). There has not been any reported in vitro characterisation of Co-EC collagen and nanocellulose.

### 3.5. Post-Alignment Fabrication Methods

In contrast to homogenous Co-EC, the second method for incorporating fillers into collagen-based scaffolds uses post-alignment fabrication methods. Due to the ease of generating threads via EC, textile-based fabrication methods have been investigated as a promising post-EC fabrication process [161]. Comparatively, the simplest fabrication method involves forming **yarn**, made by twisting several threads together. The mechanical properties of EC collagen yarn are better than that of the threads. Specifically, there was a reported 30% and 20% increase in the ultimate tensile strength (65 MPa) and Young’s modulus (530 MPa), respectively (Table 6) [143]. **Braiding** is comprised of three or more threads being intertwined in an overlapping pattern [161]. Furthermore, three individual braids can been braided again, thus, using nine individual threads [162]. This twice braided technique is suited for tissues under high load and has been used with EC collagen for tendon applications [69,152,162]. There were increases in ultimate tensile strength (24–88 MPa), strain (7–14%), and tensile modulus (277–671 MPa) when braided once and crosslinked with genipin. Additionally, braiding increased cell attachment, as the cells were able to infiltrate the space between the bundles [69]. **Weaving** involves overlapping the two distinct directions of the threads, termed warp and weft. The warp is stationary, whilst the weft is perpendicular. The weft moves in a repeating under-over fashion, forming rows [161]. Younesi et al. combined these fabrication methods by firstly forming yarn with three EC collagen threads (3-ply), and then the yarn was subsequently woven [143]. Xie et al. used polylactic acid (PLA) threads twisted around a two-ply EC collagen core yarn, which were then woven into a scaffold [151]. Both methods were used to fabricate sheets for tendon applications. The resulting scaffolds have a reported porosity of 81%. High porosity has been noted as necessary for the diffusion of oxygen, nutrients, and waste (Table 7) [163]. Finally, **knitting** is the most complicated textile fabrication method. Individual threads or yarns that are interlaced in a highly ordered arrangement of connected loops brought through a previous loop forming new rows [161]. This method has been used by Xie et al. to fabricate myocardial patches. Specifically, two continuous EC collagen threads and a PLA were grouped into yarn. The yarn was subsequently knitted and crosslinked with EDC/NHS, resulting in a maximum scaffold load of 1.4 N, an extension of 3.1 mm, and 1.8 N.mm^−1^ stiffness [34].

**The Layer-by-layer** assembly provides a simple method for reinforcing scaffolds via stacking, increasing their robustness and providing more surface area. Chen et al. layered EC collagen membranes with human corneal stromal cells attached in alternating directions of alignment to mimic the structure of corneas [35]. Nguyen et al. investigated the reinforcing of scaffolds using tubes with threads for small-diameter blood vessels [61]; EC collagen tubes and threads were fabricated, with the reinforcing threads positioned around the collagen tubular lumen in either longitudinal or circumferential directions and crosslinked with EDC/NHS. The addition of the EC collagen threads increased the overall tube scaffold strength, with the circumferentially-directed threads demonstrating better performance when compared to the longitudinally orientated version (Table 6).

### 3.6. Clinical Applications Using Electro-Compacted Collagen Scaffolds

EC collagen has been used to fabricate biomimetic scaffolds for various applications. Such applications have included the biofabrication of tendons [69], corneas [18], nerves [115], blood vessels [117], myocardium [34], and wound-healing dressings [116]. Due to the wide scope of clinical applications for EC collagen scaffolds, there has been a variety of cell types used and in vitro characterisations made; however, currently, there are limited in vivo studies available (Table 8). 

For **tendon** repair and reconstruction, Kishore et al. fabricated scaffolds and assessed the cellular [64] and implanted responses [162]. Three EC collagen threads crosslinked with genipin (length 4 cm, width 400–500 μm, and thickness 200–300 μm) were braided, and three sets of braids were again braided, resulting in nine total threads per the twice-braided scaffold. Individual threads were used to assess human mesenchymal stem cell (hMSCs) cytocompatibility and suitability for tenogenic differentiation [64,165]. Alamar blue assays demonstrated a two-fold higher cell adhesion for the EC threads (40%), when compared to the unaligned version (20%). However, cell proliferation was observed as significantly higher for unaligned threads (15-fold) compared to EC threads (5-fold). The early (scleraxis) and mature (tenomodulin) markers for tendon differentiation were significantly higher for the EC threads, promoting tenogenic differentiation. Furthermore, a specific marker (osteocalcin) for bone differentiation was greater in the unaligned threads, resulting in alignment-suppressing osteogenic differentiation. The scaffolds were implanted into the plantar tendons of female New Zealand white rabbits. The scaffolds displayed limited degradation for the first four months; from four to eight months, the scaffold size significantly decreased, whilst granulomatous inflammation also decreased, being comparable to that around the sutures (4-0 PDS, Ethicon, Cincinnati, OH, USA). The histological examination showed an inflammatory core mainly populated with macrophages and very few lymphocytes, neutrophils, or eosinophils; additionally, no foreign body giant cells were observed, demonstrating good biocompatibility [162]. Furthermore, EC collagen scaffolds implanted in the infraspinatus tendon and seeded with autologous MSCs demonstrated a comparable maximum failure load to that of the contralateral control shoulders [164].

EC collagen has been investigated for **corneal** biofabrication due to the highly transparent and mechanically robust nature of EC collagen membranes. Initially, Kishore et al. assessed the in vitro response of human keratocytes (corneal fibroblasts) on EDC/NHS crosslinked EC membranes [18]. A live-dead assay showed the high keratocyte viability on the crosslinked membranes, and F-actin staining (at day 2) demonstrated well-spread morphology and attachment; by day 7, a highly confluent layer was observed. Additionally, due to the function of corneas, scaffold transparency was investigated where light transmission measurements determined that the crosslinking reduced the scaffold’s transparency (EDC/NHS 67–89%; genipin 33–78%). However, after 14 days of culture with keratocytes, the EC collagen scaffolds (EDC/NHS) had increased in transparency by 75–100%. Meanwhile, Chen et al. aimed to fabricate a biomimetic corneal stromal structure with orthogonally aligned layers [35]. Four EC membranes seeded with human corneal stromal cells (hCSCs) were layered onto each other in alternating alignment directions, forming an orthogonally arranged scaffold. Cell orientation was investigated by F-actin staining, showing the underlying scaffold topography affecting cell alignment. Specifically, cells on the EC membranes were clearly aligned with collagen fibrils whilst conventional collagen scaffolds were patently disordered, resulting in scaffold alignment directly affecting cell orientation. The multilayered scaffold was shown to upregulate keratocyte expression (ALDH3) whilst reducing fibroblast phenotypes (α-SMA and Thy-1), confirming keratocyte differentiation from hCSCs, mimicking the quintessential state of human corneal stroma. Furthermore, there was no change in glucose permeability or the mass of the cornea scaffolds over time, whilst a small decrease in the dehydrated mass was observed at days 7 and 14, and the presence of the cells marginally impaired light transmission (81–83%). 

The effects of collagen alignment via EC were investigated for the application of **nerve** growth by Abu-Rub et al. [115]. Rat pheochromocytoma (PC12) cells were cultured on either EC threads or conventional unaligned collagen membranes, and embryonic rat dorsal root ganglion explants were subsequently placed on the collagen scaffolds (or adjacent to the aligned threads) to assess neurite extension after growth. The cells seeded onto the threads displayed outgrowth that continued in the direction of the fibre, and the unaligned scaffolds displayed no preferential neurite outgrowth, whilst the cells seeded away from the thread showed random outgrowth until contacting the thread, which then changed their trajectory to follow the orientation of the threads. It was also noted, for the first time, that cells were able to overcome myelin-associated glycoprotein-induced inhibition when on EC collagen threads, without surface modification or chemical functionalisation. 

EC collagen has been used to fabricate small-diameter **blood vessels**. Nguyen et al. initially investigated the effects of incorporating elastin into collagen threads due to elastin’s natural prevalence in the wall of blood vessels [117]. Rat aortic smooth muscle cells (rSMCs) were seeded onto collagen-only and collagen and elastin scaffolds. The Alamar blue assay (at day 1) showed that the collagen-only scaffolds displayed preferential alignment, which was not seen in the elastin-containing threads; however, by day 14, a confluent and highly aligned layer of cells was observed on both fibre types. Contractile (α-SMA and calponin) and synthetic (thrombospondin) phenotype markers were examined by PCR, where elastin-containing scaffolds showed an increased expression of α-SMA and calponin from days 3–14 whilst remaining the same on the collagen-only scaffolds. Furthermore, thrombospondin expression increased in both thread types over time, confirming that the incorporation of elastin into EC collagen induces a contractile expression in rSMCs. Further work by Nguyen et al. fabricated tube scaffolds, seeded initially with rSMCs, as previously described [61]. Additionally, human umbilical vein endothelial cells (hUVECs) were seeded to a scaffold lumen cell cytoskeleton, and staining demonstrated that the cells could successfully attach and proliferate on the luminal surface. The immunostaining of hUVECs showed evidence of gap-junction (Cx43) expression around dense colonies, confirming the presence of intercellular interactions. Furthermore, the cells were positive for nitric oxide production (eNOS) and endothelial cell phenotypes (vWF), suggesting successful endothelial cell differentiation. 

Xie et al. investigated continuous EC collagen threads as materials for textile-based fabrication methods for the application of **myocardium** [34]. The scaffolds were fabricated by grouping two collagen and one polylactic acid (PLA) or PLA-only threads together, forming yarn, and were subsequently knitted into scaffolds. The collagen-containing scaffolds, when seeded with human cardiosphere-derived cells (hCDCs), allowed for attachment, proliferation, and migration across the full surface as determined by Alamar blue assay. Whilst PLA had a limited initial biological response, the cells formed surface aggregates and attached between the adjacent yarns and were able to proliferate. On day 28, both groups were compatible, with a maintained confluence. 

Yuan et al. used a bacterial nanocellulose (BNC) scaffold impregnated with collagen and lactoferrin (LF) via EC for **wound healing** applications [159]. Five groups of dressings were investigated (BNC, BNC-LF, BNC-Col, BNC-LF-Col, and cotton gauze) by making wounds (1 cm in diameter) on the dorsal flank of male Sprague-Dawley rats, with the dressings changed daily. The groups that had collagen incorporated into the dressing showed greater healing efficiency than those without. The BNC-LF-Col scaffold showed the highest reduction in wound size after nine days at 85% and had the highest presence of fibroblasts. However, it is noteworthy that this study did not directly assess EC collagen *in vivo,* even more so, the effects of collagen and lactoferrin integration into nanocellulose scaffolds for healing. 

## 4. Conclusion and Future Directions

Collagen-based scaffolds, without modification, lack the mechanical characteristics for use in many tissues. Aligning the collagen provides a method for increasing its mechanical robustness, and each method of alignment has its associated benefits and limitations. Electro-compaction has been identified as a highly effective alignment method, which utilises a simple cost-effective setup without the use of harmful solvents. The EC of collagen has the ability to enhance scaffold strength whilst better mimicking natural tissue orientation and improving cellular activity. Currently, the largest limiting factor of EC collagen use is its insufficient mechanical strength; due to this, methods have been investigated to enhance its mechanical properties, such as PBS treatment to induce fibrogenesis, chemical crosslinking, using EDC/NHS or genipin, and post-alignment fabrication methods. Additionally, the ability to add filler materials via co-electro-compaction can enhance the natural properties of collagen, such as increasing the robustness of the scaffold and the induction of specialised biological phenotypes. EC collagen is being researched for a wide range of clinical applications, such as corneal, muscle, tendon, nerve, blood vessel, myocardium, and skin biofabrication. Future work can combine the incorporation of tissue-specific collagen types and naturally occurring extracellular matrix components as filler materials. Specifically, proteins, such as elastin, fibronectin, and laminin or proteoglycans, such as glycosaminoglycans, heparin sulphate, and chondroitin sulphate, can be used to fabricate biomimetic structures. Furthermore, new fabrication methods are required to better mimic the complex three-dimensional tissue structures whilst maintaining fibre direction. Finally, there have been limited in vivo studies investigating the effects of implanted EC collagen-based scaffolds, which will be required for clinical translation.

## Figures and Tables

**Table 1:** Classification of collagen types.

Collagen Classification	Collagen Type	Distribution
Fibril-forming	I	Bone, skin, tendon, ligament, cornea
	II	Cartilage, vitreous humour
	III	Skin, blood vessel
	V	Bone, dermis
	XI	Cartilage, intervertebral disc
	XXIV	Bone, cornea
	XXVII	Cartilage
FACIT ^1^	VII	Bladder, dermis
	IX	Cartilage, cornea
	XII	Tendon, dermis
	XIV	Bone, dermis, cartilage
	XVI	Kidney, dermis
	XIX	Human rhabdomyosarcoma
	XX	Cornea of chick
	XXI	Kidney, stomach
	XXII	Muscle-tendon junction
	XXVI	Ovary, testis
Network forming	IV	Basement membrane
	VI	Muscle, dermis, cornea, cartilage
	VIII	Brain, skin, kidney, heart
	X	Hypertrophic cartilage
	XXVIII	Dermis, sciatic nerve
MACIT ^2^	XIII	Dermis, eye, endothelial cell
	XVII	Hemi desmosomes in epithelia
	XXIII	Heart, retina
	XXV	Heart, testis, brain
MULTIPLEXINs ^3^	XV	Capillaries, testis, kidney, heart
	XVIII	Liver, basement membrane

^1^ Fibril-Associated Collagens with Interrupted Triple-helices, ^2^ Membrane-Associated Collagens with Interrupted Triple-helices, ^3^ Multiple triple-helix domains and interruptions [21,22,23,24,25,26].

**Table 2:** Summary of collagen alignment techniques.

Method	Alignment	Collagen Concentration (mg.mL^−1^)	Effect on Collagen Structure	Mechanical Properties	Ease of Processing
Gravity-based Fluidic	++	6–14	+	Not reported	+++
Extrusion-based Fluidic	++	>15	+	Not reported	++
Stress-induced self-alignment	+	Not applicable	+	Not reported	+
Static magnet	+++	<5	+	MPa	++
Flow-magnetic	++	<5	-	MPa	++
Electrospinning	++	>50	-	MPa	++
Electro-compaction	+++	<5	+	MPa	+++

Key: + Minor improvement, ++ Medium improvement, +++ Major improvement, and - Negative effect.

**Table 3:** Summary of the mechanical properties of electro-compacted collagen scaffolds.

Sample State	Testing Method	Load Cell	Strain Rate	Young’s Modulus (MPa)	Ultimate Tensile Strength (MPa)	Ultimate Tensile Strain (%)	Ref
**Thread**							
Hydrated	Monotonic Tensile	250 N	10 mm.min^−1^	0.1–750	0.1–55	11–100	[122,143,148,149]
Dehydrated	Monotonic Tensile	250 g	10 mm.min^−1^	200–1000	10–70	3–15	[148]
**Membrane**							
Hydrated	Monotonic Tensile		0.1 N.min^−1^	4 kPa–2	10–200 kPa	10–70	[18]
Hydrated	Monotonic Tensile	10 N	10 mm.min^−1^	0.25	3.5	0.2	[116]
Hydrated	Compression		1%.s^−1^	100 kPa	35 kPa	30	[120]
Hydrated	Tensile		1%.s^−1^	30	3	30	[120]
Dehydrated	Nanoindentation			0.10–0.22 GPa			[150]
Hydrated	Rheology	Strain 1%, frequency 0.01–100 Hz, strain sweep		G′ 200–500 G″ 50–70			[35]
Hydrated	Microindentation	10 N	0.1 mm.min^−1^	0.23 kPa			[35]
Dehydrated	Hertzian Model			180–240			[126]
Dehydrated	Oliver-Pharr Model			80–130			[126]
**Tube**							
Hydrated	Monotonic Tensile Static ring		0.1 N.min^−1^	0.05–0.18	0.05–0.18	20–25	[61]
Hydrated	Rheology Cyclic	Strain 8%, frequency 1 hz, 6 sweeps, five oscillations per cycle		G′ 0.06 G″ 0.01			[61]

**Table 4:** Summary of crosslinking protocols used for collagen electro-compaction.

Crosslinker	Solvent	Exposure Concentration	Time	Temperature	Ref
EDC/NHS	50 mM MES	20 mM EDC, 20 mM NHS	4 h	Room	[116]
EDC/NHS	50 mM MES in Ethanol 70% (pH = 5.5)	10 mM EDC, 5 mM NHS	4 h	Room	[18,117]
EDC/NHS	Ethanol 80%	1:25:50 (Col:EDC:NHS)	2 h		[34,151]
EDC/NHS	Ethanol 80%	1:100:250 (Col:EDC:NHS)	15 min		[114]
Genipin	Ethanol 0, 70, 80, 80, and 100%	0, 0.1, 0.625, 2.00 and 6.00%	6, 12, 24 and 72 h	37 °C	[148]
Genipin	1 × PBS	0.625%	72 h	37 °C	[111,152]
Genipin	Ethanol 90%	0.625%	24 h	Room	[18]
Genipin	Ethanol 90%	0.625%	72 h	37 °C	[120,127,143]
Genipin	Ethanol 90%	0.625%	72 h		[31,64]

**Table 5:** Summary table of the fillers utilised for collagen-based co-electro-compaction.

Scaffold Shape	Scaffold Filler	Young’s Modulus (MPa)	Ultimate Tensile Strength (MPa)	Ultimate Tensile Strain (%)	In Vitro Response	Ref
Thread	Collagen only	10	0.4	65		[117]
Thread	Soluble elastin	3	0.2	60	+	
Thread	Insoluble elastin	4	0.2	45	+	
Thread	t-CNC	91.5–231.9	10.1–22.4	10.7–15.1	Nil	[31]
Key: t-CNC TEMPO oxidised cellulose nanocrystals, + Enhanced response compared to collagen only						

**Table 6:** Summary of the mechanical properties of post-electro-compaction fabrication methods.

Fabrication Method	Young’s Modulus (MPa)	Ultimate Tensile Strength (MPa)	Ultimate Tensile Strain (%)	Ref
Yarn	520	65	20	[143]
Braid	277–671	24–88	7–24	[152]
Lumen and cir thread	0.282	0.047	51.2	[61]
Lumen and long thread	0.114	0.024	38.3	

Key: Cir Circumferential, Long Longitudinal.

**Table 7:** Summary of maximum load, extension, and stiffness of post-electro-compaction fabrication methods.

Fabrication Method	Max Load (N)	Extension (mm)	Stiffness (N.mm^−1^)	Ref
Weave	100–350	5–10	25–89	[143,151]
Knit	1.4	3.1	1.8	[34]

**Table 8:** Summary of electro-compacted collagen scaffold applications.

Application	Cell		In Vivo	Ref
	Source	Type		
Cornea	Human	Corneal Stromal		[35]
	Human	Keratocyte		[18]
	Mouse	Corneal Stromal		[150]
Muscle	Chicken	Cardiomyocyte Skeletal Muscle		[114]
Tendon	Human	Mesenchymal Stem		[64,148]
	Rat	Mesenchymal Stem		[152]
		Tendon fibroblast		[69,152]
		Rotator cuff and Achilles tendon		[151]
			Rabbit White New Zealand	[162,164]
				[149]
Nerve	Rat	Pheochromocytoma		[115]
Blood Vessel	Rat	Aortic Smooth Muscle		[117]
	Human	Umbilical Vein Endothelial		[61,117,144]
Skin	Human	Dermal Fibroblast		[116]
	Mouse	Dermal Fibroblast	Rat Sprague-Dawley	[159]
Myocardium	Human	Cardiosphere-derived		[34]
Tissue engineering	Human	Mesenchymal Stem		[120]

## Data Availability

The data presented in this study are available on request from the corresponding author.
